# Local active information storage as a tool to understand distributed neural information processing

**DOI:** 10.3389/fninf.2014.00001

**Published:** 2014-01-28

**Authors:** Michael Wibral, Joseph T. Lizier, Sebastian Vögler, Viola Priesemann, Ralf Galuske

**Affiliations:** ^1^MEG Unit, Brain Imaging Center, Goethe UniversityFrankfurt am Main, Germany; ^2^CSIRO Computational InformaticsMarsfield, NSW, Australia; ^3^Fakultät für Biologie, Technische UniverstätDarmstadt, Germany; ^4^Department of Nonlinear Dynamics, Max Planck Institute for Dynamics and Self-OrganizationGöttingen, Germany

**Keywords:** visual system, neural dynamics, predictive coding, local information dynamics, voltage sensitive dye imaging, distributed computation, complex systems, information storage

## Abstract

Every act of information processing can in principle be decomposed into the component operations of information storage, transfer, and modification. Yet, while this is easily done for today's digital computers, the application of these concepts to neural information processing was hampered by the lack of proper mathematical definitions of these operations on information. Recently, definitions were given for the dynamics of these information processing operations on a local scale in space and time in a distributed system, and the specific concept of local active information storage was successfully applied to the analysis and optimization of artificial neural systems. However, no attempt to measure the space-time dynamics of local active information storage in neural data has been made to date. Here we measure local active information storage on a local scale in time and space in voltage sensitive dye imaging data from area 18 of the cat. We show that storage reflects neural properties such as stimulus preferences and surprise upon unexpected stimulus change, and in area 18 reflects the abstract concept of an ongoing stimulus despite the locally random nature of this stimulus. We suggest that LAIS will be a useful quantity to test theories of cortical function, such as predictive coding.

## 1. Introduction

It is commonplace to state that brains exist to “process information.” Curiously enough, however, it is much more difficult to exactly quantify this putative processing of information. In contrast, we have no difficulties to quantify information processing in a digital computer, e.g., in terms of the information stored on its hard disk, or the amount of information transferred per second from its hard disk to its random access memory, and then on to the CPU. Why then is it so difficult to perform a similar quantification for biological, and especially neural information processing?

One answer to this question is the conceptual difference between a digital computer and a neural system: in a digital computer all components are laid out such that they only perform specific operations on information: a hard disk should store information, and not modify it, while the CPU should quickly modify the incoming information and then immediately forget about it, and system buses exist solely to transfer information. In contrast, in neural systems it is safe to assume that each element of the system (each neuron) *simultaneously* stores, transfers and modifies information in variable amounts, and the component processes are hard to separate quantitatively. Thus, while in digital computers the distinction between information storage, transfer and modification comes practically for free, in neural systems separating the components of distributed information processing requires thorough mathematical definitions of information storage, transfer and modification. Such definitions, let alone a conceptual understanding of what the terms meant in distributed information processing, were unavailable until very recently (Langton, 1990; Mitchell, 1998; Lizier, 2013).

These necessary mathematical definitions were recently derived building on Turing's old idea that every act of information processing can be decomposed into the component processes of information storage, transfer and modification (Turing, 1936)—very much in line with our everyday view of the subject. Later, Langton and others expanded Turing's concepts to describe the emergence of the capacity to perform arbitrary information processing algorithms, or “universal computation,” in complex systems, such as cellular automata (Langton, 1990; Mitchell et al., 1993), or neural systems. The definitions of information transfer and storage were then given by Schreiber (2000), Crutchfield and Feldman (2003), and Lizier et al. (2012b). However, the definition of information modification is still a matter of debate (Lizier et al., 2013).

Of these three component processes above—information transfer, storage, and modification—information storage in particular has been used with great success to analyze cerebro-vascular dynamics (Faes et al., 2013), information processing in swarms (Wang et al., 2012), and most importantly, to evolve (Prokopenko et al., 2006), and optimize (Dasgupta et al., 2013) artificial information processing systems. This suggests that the analysis of information storage could also be very useful for the analysis of neural systems.

Yet, while neuroscientists have given much attention to considering how information is stored structurally in the brain, e.g., via synaptic plasticity, the same attention has not been given to information storage in neural dynamics, and its quantification. As an exception Zipser et al. (1993) clearly contrasted two different ways of storing information: *passive storage*, where information is stored “in modified values of physiological parameters such as synaptic strength,” and *active storage* where “information is preserved by maintaining neural activity throughout the time it must be remembered.” In the same paper, the authors go on to point out that there is evidence for the use of both storage strategies in higher animals, and link the relatively short time scale for active storage (at maximum in the tens of seconds) with short-term or working memory and, therefore, refer to it as “active information storage.”

Despite the importance of information storage for neural information processing, information theoretic measures of active information storage have not yet been used to quantify information processing in neural systems, and in particular not to measure spatiotemporal patterns of information storage dynamics. Therefore, it is the aim of this article to introduce measures of information storage as analysis tools for the investigation of neural systems, and to demonstrate how cortical information storage in visual cortex unfolds in space and time. We will also demonstrate how neural activity may be misinformative about its own future and thereby generates “surprise.”

To this end, we first give a rigorous mathematical definition of information storage in dynamic activity in the form of local active information storage (LAIS). We then show how to apply this measure to voltage sensitive dye imaging data from cat visual cortex. In these data, we found sustained increases in dynamic information storage during visual stimulation, organized in clear spatiotemporal patterns of storage across the cortex, including stimulus-specific spatial patterns, and negative storage, or surprise, upon a change of the stimulus. Finally, we discuss the implications of the LAIS measure for neurophysiological theories of predictive coding [see Bastos et al. (2012), and references therein], that have been suggested to explain general operating principles of the cortex and other hierarchical neural systems.

## 2. Materials and methods

The use of the stored information for information processing inevitably requires its re-expression in neural activity and its interaction with ongoing neural activity and incoming information. Hence, information storage *actively in use for information processing* will inevitably be reflected in the dynamics of neural activity, and is therefore accessible in recordings of neural activity alone. To quantify this stored information that is present in neural time series we will now introduce a measure of information storage called *local active information storage* (Lizier et al., 2012b). In brief, this measure quantifies the amount of information in a sample from a neural time series that is predictable from its past—and thereby has been stored in this past. This is done by simply computing the local mutual information between the past of a neural signal and its next sample at each point in time, and for each channel of a recording. As the following material is necessarily formal, the reader may consider skipping ahead to section 2.2.3 at first reading to gain an intuitive understanding of mechanisms that serve active information storage.

### 2.1. Notation and information theoretic preliminaries

To avoid confusion, we first have to state how we formalize observations from neural systems mathematically. We define that a neural (sub-)system of interest (e.g., a neuron, or brain area) 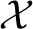 produces an observed time series {*x*_1_, …, *x*_*t*_, …, *x*_*N*_}, sampled at time intervals δ. For simplicity we choose our temporal units such that δ = 1, and hence index our measurements by *t* ϵ {1…*N*} ⊆ ℕ, i.e., we index in terms of samples. The full time series is understood as a realization of a *random process* X. This random processes is nothing but a collection of random variables *X*_*t*_, sorted by an integer index (*t* in our case). Each random variable *X*_*t*_, at a specific time *t*, is described by the set of all its *J* possible outcomes 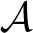
_*X*_*t*__ = {*a*_1_, …, *a*_*j*_, …, *a*_*J*_}, and their associated probabilities *p*_*t*_(*x*_*t*_ = *a*_*j*_). The probabilities of a specific outcome *p*_*t*_(*x*_*t*_ = *a*) may change with *t*, i.e., when going from one random variable to the next. In this case, we will indicate the specific random variable *X*_*t*_ the probability distribution belongs to—hence the subscript in *p*_*t*_(·). For practical estimation of *p*_*t*_(·) then, multiple time-series realizations or trials would be required. For stationary processes, where *p*_*t*_(*x*_*t*_ = *a*) does not change with *t*, we simply write *p*(*x*_*t*_), and practical estimation may be done from a single time-series realization. In sum, in this notation the individual random variables *X*_*t*_ produce realizations *x*_*t*_, and the time-point index of a random variable *X*_*t*_ is necessary when the random process is non-stationary. When using more than one system, the notation is generalized to multiple systems 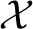, 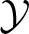, 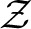, ….

As we will see below, active information storage is nothing but a specific mutual information between collections of random variables in the process in question. We therefore start by giving the definition of *mutual information* (MI) *I*(*X*; *Y*) as the amount of information held in common by two random variables *U*, *V* on average (Cover and Thomas, 1991):


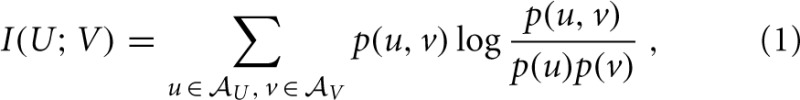



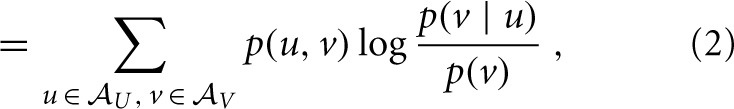


where the log can be taken to an arbitrary base, and choosing base 2 yields the mutual information in bits. Note that the mutual information *I*(*U*; *V*) is symmetric in *U* and *V*. As shown more explicitly in Equation (2), the MI *I*(*U*; *V*) measures the amount of information provided (or the amount that uncertainty is reduced) by an observation of a specific outcome *u* of the variable *U* about the occurrence of another specific outcome *v* of *V*—on average over all possible values of *u* and *v*. As originally pointed out by Fano (1961), the summands $\log\frac{p(v|u)}{p(v)}$ have a proper interpretation even without the weighted averaging—as the information that observation of a specific *u* provides about the occurrence of a specific *v*. The *pointwise* or *local mutual information* is therefore defined as:

(3) $$i(u;\;v)=\log\frac{p(v|u)}{p(v)}.$$

It is important to note the distinction of the local mutual information measure *i*(*x*; *y*) considered here from partial localization expressions, i.e., the partial mutual information or specific information *I*(*u*; *V*) which are better known in neuroscience (DeWeese and Meister, 1999; Butts, 2003; Butts and Goldman, 2006). Partial MI expressions consider information contained in specific values *u* of one variable *U* about the other (unknown) variable *V*. Crucially, there are two valid approaches to measuring partial mutual information, one which preserves the additivity of information and one which retains non-negativity (DeWeese and Meister, 1999). In contrast, the fully local mutual information *i*(*x*; *y*) that is used here is uniquely defined as shown by Fano (1961).

### 2.2. Local active information storage

Using the definition in Equation (3), we can immediately quantify how much of the information in the outcome *x*_*t*_ of the random variable *X*_*t*_ at time *t* was predictable from the observed past *state* **x**^*k*−^_*t* − 1_ of the process at time *t* − 1:

(4) $$a(x_{t})=i(x_{t\;-\;1}^{k-};\;x_{t})$$

(5) $$=\log\frac{p_{t}(x_{t}|x_{t\;-\;1}^{k-})}{p_{t}(x_{t})}.$$

This quantity was introduced by Lizier et al. (2012b) and called *local active information storage* (LAIS). Here, **x**^k−^_*t* − 1_ is an outcome of the collection of previous random variables **X**^*k*−^_*t* − 1_ = {*X*_*t* − 1_, *X*_*t* − *t*_1__, …, *X*_*t* − *t*_*k*_max___}, called a *state* (see below). The corresponding expectation value over all possible observations of *x*_*t*_ and **x**^*k*−^_*t* − 1_, *A*(*X*_*t*_) = *I*(**X**^*k*−^_*t* − 1_; *X*_*t*_), is known simply as the *active information storage*. The naming of this measure aligns well with the concept of active storage in neuroscience by Zipser et al. (1993), but is more general than capturing only sustained firing patterns. In the following subsections, we comment on practical issues involved in estimating the LAIS, and discuss its interpretation.

#### 2.2.1. Interpretation and construction of the past state

As indicated above, the joint variable **x**^*k*−^_*t* − 1_ in Equation (4) is an outcome of the collection of previous random variables: **X**^*k*−^_*t* − 1_ = {*X*_*t* − 1_, *X*_*t* − *t*_1__,…, *X*_*t* − *t*_*k*_max___}. This collection should be constructed such, that it captures the *state* of the underlying dynamical system 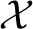, and can be viewed as a state-space reconstruction of this system. In this sense, **X**^*k*−^_*t* − 1_ must be chosen such that *X*_*t*_ is conditionally independent of all *X*_*t* − *t*_*l*__ with *t*_*l*_ > *t*_*k*_max__, i.e., of all variables that are observed earlier in the process X than the variables in the state at *t* − 1. The choice must be made carefully, since using too few variables *X*_*t* − *t*_*l*__ from the history can result in an underestimation of *a*(*x*_*t*_), while using too many [given the amount of data used to estimate the probability density functions (PDFs) in Equation (4)] will artificially inflate it. Typically, the state can be captured via Takens delay embedding (Takens, 1981), using *d* variables *X*_*t* − *t*_*l*__ with the *t*_*l*_ delays equally spaced by some τ ≥ 1, with *d* and τ selected using the Ragwitz criteria (Ragwitz and Kantz, 2002)—as recommended by Vicente et al. (2011) for the related transfer entropy measure (Schreiber, 2000). Alternatively, non-uniform embeddings may be used (e.g., see Faes et al., 2012).

If the process has infinite memory, and *k*_max_ does not exist, then the local active information storage is defined as the limit $\underset{k\to \infty }{\lim}$ of Equation (4):

(6) $$a(x_{t})=\underset{k\to \infty }{\lim}i(x_{t\;-\;1}^{k-};\;x_{t})$$

(7) $$=\underset{k\to \infty }{\lim}\log\frac{p_{t}(x_{t}|x_{t\;-\;1}^{k-})}{p_{t}(x_{t})}.$$

#### 2.2.2. Relation to other measures and dynamic state updates

The *average* active information storage (AIS), is related to two measures introduced previously. On the one hand, a similar measure called “regularity” had been introduced by Porta et al. (2000). On the other hand, AIS is closely related to the excess entropy (Crutchfield and Feldman, 2003), as observed in Lizier et al. (2012b). The excess entropy *E*(*X*_*t*_) = *I*(**X**^*k*−^_*t* − 1_; **X**^*k*+^_*t*_), with **X**^*k*+^_*t*_ = {*X*_*t*_, *X*_*t* + *t*_1__, …, *X*_*t* + *t*_*k*_max___} being a similar collection of future random variables from the process, measures the amount of information (on average) in the future outcomes **x**^*k*+^_*t*_ of the process this is predictable from the observed past state **x**^*k*−^_*t* − 1_ at time *t* − 1. As such, the excess entropy captures all of the information in the future of the process that is predictable from its past. In measuring the subset of that information in only the next outcome of the process, the AIS is focused on the dynamic state updates of the process.

From the point of view of dynamic state updates, the AIS is *complementary* to a well-known measure of uncertainty of the next outcome of the process which cannot be resolved by its past state. Following Crutchfield and Feldman (2003) we refer to this quantity as the “entropy rate,” the conditional entropy of the next outcome given the past state: *H*_μ_(*X*_*t*_) = *H*(*X*_*t*_ | **X**^*k*−^_*t* − 1_) = 〈− log_2_*p*_*t*_(*x*_*t*_ | **x**^*k*−^_*t* − 1_)〉. The complementarity of the entropy rate and AIS was shown by Lizier et al. (2012b): *H*(*X*_*t*_) = *A*(*X*_*t*_) + *H*_μ_(*X*_*t*_), where *H*(*X*_*t*_) is the Shannon entropy of the next measurement *X*_*t*_. *H*_μ_(*X*_*t*_) is approximated by measures known as the Approximate Entropy (Pincus, 1991), Sample Entropy (Richman and Moorman, 2000), and Corrected Conditional Entropy (Porta et al., 1998), which have been well studied in neuroscience [see e.g., the work by Gómez and Hornero (2010); Vakorin et al. (2011), and references therein]. Many such studies refer to *H*_μ_(*X*_*t*_) as a measure of complexity, however, modern complex systems perspectives focus on complexity as being captured in how much structure can be resolved rather than how much cannot (Crutchfield and Feldman, 2003).

Furthermore, given that the most appropriate measure of complexity of a process is a matter of open debate (Prokopenko et al., 2009), we take the perspective that complexity of a system is best approached as arising out of the interaction of the component operations of information processing: information storage, transfer and modification (Lizier, 2013), and focus on measuring these quantities since they are rigorously defined and well-understood. Crucially, in comparison to the excess entropy discussed above, the focus of AIS in measuring the information storage in use in dynamic state updates of the process make it directly comparable with measures of information transfer and modification. Of particular importance here is the relationship of AIS to the transfer entropy (Schreiber, 2000), where the two measures together reveal the sources of information (either being the past of that process itself—storage, or of other processes—transfer) which contribute to prediction of the process' next outcome.

The formulation of the transfer entropy specifically eliminates information storage in the past of the target process from being mistakenly considered as having been transferred (Lizier and Prokopenko, 2010; Lizier, 2013; Wibral et al., 2013). An interesting example is where a periodic target process is in fact causally driven by another periodic process—after any initial entrainment period, our information processing view concludes that we have information storage here in the target but no transfer from the driver (Lizier and Prokopenko, 2010). While causally there is a different conclusion, our observational information processing perspective is simply focussed on decomposing apparent information sources of the process, regardless of underlying causality (which in practise cannot often be determined anyway). In this view, a causal interaction can computationally subserve both information storage or transfer (as discussed further in the next section). Information transfer is necessarily linked to a causal interaction, but the reverse is not true. It has previously been demonstrated that the information processing perspective is more relevant to emergent information processing structure in complex systems, e.g., coherent information cascades, in contrast to causal interactions being more relevant to the micro-scale physical structure of a system, e.g., axons in a neural system (Lizier and Prokopenko, 2010).

#### 2.2.3. Mechanisms producing active information storage

In contrast to passive storage in terms of modifications to system structure (e.g., synaptic gain changes), the mechanisms underlying active information storage are not immediately obvious. The mechanisms that subserve this task have been formally established, however, and can be grouped as follows:

1. *Physical mechanisms in the system*. This could incorporate some internal memory mechanism in the individual physical element giving rise to the process X (e.g., some decay function, or the stereotypical processes during the refractory period after a neural spike). More generally, it may involve network structures which offload or distribute the memory function onto edges or other nodes. In particular, Zipser et al. (1993) reported that networks with fixed, *recurrent connections* were sufficient to account for such active storage patterns, which is in line with earlier proposals. Furthermore, Lizier et al. (2012a) quantified the AIS contribution from self-loops, feedback and feedforward loops (as the only network structures contributing to active information storage).
2. *Input-driven storage*. This describes situations where the apparent memory in the process is caused by information storage structure which lies in another element which is driving that process, e.g., a periodically spiking neuron that may cause a downstream neuron to spike with the same period (Obst et al., 2013). As described in section 2.2.2 above, an observer of the process attributes these dynamics to information storage, regardless of the (unobserved) underlying causal mechanism.

Of these mechanisms of active information storage the case of circular causal interactions in a loop motif, and the causal, but repetitive influence from another part of the system may seem counterintuitive at first, as we might think that in these cases there should be information transfer rather than active information storage. To see why these interactions serve storage rather than transfer, it may help to consider that *all* components of information processing, i.e., transfer, active storage and modification, ultimately have to rely on causal interactions in physical systems. Hence, the presence of a causal interaction cannot be linked in a one-to-one fashion to information transfer, as otherwise there would be no possibility for physical causes of active information storage and of information modification left, and no consistent decomposition of information processing would be possible. Therefore, the notion of storage that is measurable in a part of the system but that can be related to external influences onto that part is to be preferred for the sake of mathematical consistency and ultimately, usefulness. We acknowledge that information transfer has often been used as a proxy for a causal influence, dating back to suggestions by Wiener (1956) and Granger (1969). However, now that causal interventional measures and measures of information transfer can be clearly distinguished (Ay and Polani, 2008; Lizier and Prokopenko, 2010) it seems no longer warranted to map causal interactions to information transfer in a one-to-one manner.

#### 2.2.4. Interpretation of LAIS values

Measurements of the LAIS tells us the amount to which observing the past *state* **x**^*k*−^_*t* − 1_ reduced our uncertainty about the specific next outcome *x*_*t*_ that was observed. We can interpret this in terms of *encoding* the outcome *x*_*t*_ in bits: encoding *x*_*t*_ using an optimal encoding scheme for the distribution *p*_*t*_(*x*_*t*_) takes −log_2_*p*_*t*_(*x*_*t*_) bits, whereas encoding *x*_*t*_ if we know **x**^*k*−^_*t* − 1_ using an optimal encoding scheme for the distribution *p*_*t*_(*x*_*t*_ | **x**^*k*−^_*t* − 1_) takes −log_2_*p*_*t*_(*x*_*t*_ | **x**^*k*−^_*t* − 1_) bits, and the LAIS is the number of bits saved via the latter approach.

At first glance we may assume that the LAIS is a positive quantity. Indeed, as a mutual information, the *average* AIS will always be non-negative. However, the LAIS can be negative as well as positive. It is positive where *p*_*t*_(*x*_*t*_ | **x**^*k*−^_*t* − 1_) > *p*_*t*_(*x*_*t*_), i.e., where the observed past state **x**^*k*−^_*t* − 1_ made the following observation *x*_*t*_ more likely to occur than we would have guessed without the knowledge of the past state. In this case, we state that **x**^*k*−^_*t* − 1_ was informative. In contrast, the LAIS is negative where *p*_*t*_(*x*_*t*_ | **x**^*k*−^_*t* − 1_) < *p*_*t*_(*x*_*t*_); i.e., where the observed past state **x**^*k*−^_*t* − 1_ made the following observation *x*_*t*_ less likely to occur than we would have guessed without the knowledge of the past state (but it occurred nevertheless, making the cue given by **x**^*k*−^_*t* − 1_ misleading). In this case, we state that **x**^*k*−^_*t* − 1_ was *misinformative* about *x*_*t*_. To better understand negative LAIS also see the further discussion in Lizier et al. (2012a), including examples in cellular automata where the past state of a variable was misinformative about the next observation due to the strong influence of an unobserved other source variable at that time point.

#### 2.2.5. Choice of the overall time window for constructing probability densities from data

As already pointed out above, active information storage is tightly related to predictability of a given brain area's output as seen by the receiving brain area. This predictability hinges on the ability of the receiver to see the past states in the output of a brain area (see previous section) and to interpret the past states in the received time series in order to make a prediction about the next value. In other words, the receiver needs to guess *p*_*t*_(*x*_*t*_, **x**^*k*−^_*t* − 1_) correctly in order to exploit the active information storage. If the guess of the receiving neuron (*n*) or brain area, i.e., $\tilde{p_{n}}$(*x*_*t*_, **x**^*k*−^_*t* − 1_), is incorrect, then only a fraction of the information storage can be used for successfully predicting future events. The losses could be quantified as the extra coding cost for the receiving area, when assuming $\tilde{p_{n}}$(·) instead of *p*_*t*_(·). This loss would simply be the Kullback–Leibler divergence *D*_KL_(*p*_*t*_||$\tilde{p_{n}}$). This scenario sees the receiving brain area mostly as an optimal encoder or compressor. In contrast, the cost occurring in the framework of predictive coding theories would arise because the receiving brain area could not predict the incoming signal well, and thereby inhibit it via feedback to the sending brain area (Rao and Ballard, 1999). In this scenario, the cost of imperfect predictions resulting from using $\tilde{p_{n}}$ instead of *p*_*t*_, would be reduced inhibition and a more frequent signaling of prediction errors by the sending system, leading to a metabolic cost.

To see the storage that the receiving brain area can exploit, the time interval used for the practical estimation of the probability density functions (PDFs) from neural recordings should best match the expected sampling strategy of the receiving brain area. For example, if we think that probabilities are evaluated over long time frames, then it might make sense to pool all available data in the experiment, as even a mis-estimation of the true probability densities *p*_*t*_(·) (due to potential non-stationarities) then will better reflect the internal estimate $\tilde{p_{n}}$(*x*_*t*_, **x**^*k*−^_*t* − 1_), and thus the *internally* predictable information. However, if we think that probabilities are only estimated instantaneously by pooling over all available inputs to a brain area at any time point, then we should construct the necessary PDFs only from all simultaneously acquired data from all measurement channels, but not pool over time. The latter view could also be described as assuming that the brain area receiving the signals in question computes the PDF instantaneously by pooling over all its inputs, without keeping any longer term memory of the observed probabilities. This construction of a PDF would be linked closely to an instantaneous physical ensemble approach, considering that all incoming channels are physically equivalent, but are only assessed at a single instant in time. In contrast, if we assume that learning of the relevant PDFs takes place on a lifelong timescale, then PDFs should be acquired from very long recordings of a freely behaving subject or animal in a natural environment, and the outcomes of a specific experiment should be interpreted using this “lifelong” PDF. Here we lean toward this latter approach and pool all available data to estimate the internally available $\tilde{p_{n}}$.

Note that while we indeed pool over all the available data to obtain the distribution $\tilde{p_{n}}$, the interpretation of the data in terms of the active information storage is *local per agent and time step*. This is exactly the meaning of “local” in local active information storage as introduced in Lizier et al. (2012b) (this is also akin to the relation of the local mutual information introduced by Fano (1961) and the corresponding global PDF). The local active information storage values are thus obtained by interpreting realizations for a single agent and a single time step in the light of a probability distribution that is obtained over a more global view of the system in space and time. This is also indicated by the use of $\tilde{p_{n}}$ instead of *p*_*t*_. Also see the discussion section for potential other choices of obtaining *p*.

### 2.3. Acquisition of neural data

#### 2.3.1. Animal preparation

Data were obtained from an anesthetized cat. The animal had been anesthetized and artificially ventilated with a mixture of O_2_ and N_2_O (30/70%) supplemented with Halothane (0.7%). All procedures were along the guidelines of the Society for Neuroscience, in accordance with the German law for the protection of laboratory animals, permitted by the local authorities and overseen by a designated veterinarian.

#### 2.3.2. Voltage sensitive dye imaging

For optical imaging the visual cortex (area 18) was exposed and an imaging chamber was implanted over the craniotomy. The chamber was filled with silicone oil and sealed with a glass plate. A voltage sensitive dye (RH1691, Optical Imaging Ltd, Rehovot, Israel) was applied to the cortex for about 2 h and subsequently the excess of the dye was washed out. For imaging we used a CMOS camera system (Imager 3001, Optical Imaging Ltd, Rehovot, Israel, Camera: Photon Focus MV1 D1312, chip size 1312 × 1082 pixel) fitted with a lens system consisting of two 50 mm Nikon objectives providing a field of view of 8.7 × 10.5 mm and an epifluorescence illumination system (excitation: 630 ± 10 nm, emission high pass 665 nm). In order to optimize the signal-to-noise ratio raw camera signals were spatially binned to 32 × 32 camera pixels allowing for a spatial resolution of 30 × 32 μm^2^ per data pixel. Camera frames were collected at a rate 150 Hz, resulting in a temporal resolution of 6.7 ms.

#### 2.3.3. Visual stimulation

Stimuli were presented triggered to the heartbeat of the animal for 2 s and camera frames were collected during the entire stimulation period. We will denote such a single stimulation period and the corresponding data acquisition as a trial here. Each trial consisted of 1 s stimulation with an isoluminant gray screen followed by stimulation with fields of randomly positioned dots (dot size: 0.23° visual angle; 384 dots distributed over an area of 30° (vertical) by 40° (horizontal) visual angle) moving coherently in one of eight different directions at 16 degree/s. Stimuli were presented in blocks of 16 trials, consisting of eight trials using the stimuli described before and an additional eight trials which consisted only of the presentation of the isoluminant gray screen for 2 s (“blank trials”). Each motion direction condition was presented eight times in total (eight trials), resulting in the presentation on 64 stimulus trials and 64 blank trials in total. Of the presented set of eight stimulus types, seven were used for the final analysis, as the computational process for one condition did not finish on time before local compute clusters were taken down for service.

#### 2.3.4. VSD data post-processing

After spatial binning of 32 × 32 camera pixels into one data pixel, VSD data were averaged over all presentations of blank trials and this average was subtracted from the raw data to remove the effects of dye-bleaching and heartbeat. Finally, the data were denoised using a median filter of 3 × 3 data pixels.

### 2.4. Measurement of LAIS on VSD neural data

Estimation of LAIS was performed using the open source *Java information dynamics toolkit* (JIDT) (Lizier, 2012), with a history parameter *k*_max_ of ten time points, spaced 2 samples, or (2/150 Hz)= 13.3 ms, apart. The total history length thus covered 133 ms, or roughly one cycle of a neural theta oscillation, which seems to be a reasonable time horizon for a downstream neural population that ultimately must assess these states. To enable LAIS estimation from a sufficient amount of samples, we considered the data pixels as homogeneous variables executing comparable state transitions, such that the pixels form a physical ensemble in terms of information storage dynamics. Pooling data over pixels thus enables an ensemble estimate of the PDFs in question. This approach seems justified as all pixels reported activity from a single brain area (area 18 of cat visual cortex, see below). Mutual information was estimated using a box kernel-estimator (Kantz and Schreiber, 2003) with a kernel width of 0.5 standard deviations of the data.

Here we assume that the neural system is at least capable of exploiting the statistics arising from the stimulation given throughout the experiment and thus construct PDFs from all data (time points and pixels) for a given condition. Therefore, we pool data over the full time course from −1 to 1 s of the experiment. Thus, each image of the VSD data had a spatial configuration of 67 × 137 spatial data pixels after removal of the two rows/columns on each side of an image because of the median filter that was applied. Each trial (of a total of eight trials per condition) resulted in 288 LAIS values, based on an original data length of 298 samples and a history length (state dimension) of 10 pixels. The product of final image size and LAIS samples resulted in 2.64 · 10^6^ data points per trial for the estimation of the PDF for each of the eight motion direction conditions. Due to computational limitations, LAIS estimates were performed on two blocks of four trials separately, resulting in 1.06 · 10^7^ data points entering the estimation in JIDT.

### 2.5. Correlation analysis of LAIS and VSD data

For each of the seven analyzed motion direction conditions, VSD data and LAIS were initially organized separately per condition into 5 dimensional data structures, with dimensions: blocks (1,2), trials (1–4), time (−1 to 1 s), and pixel row (67) and columns (137). For correlation analysis, these arrays were linearized and entered into a Spearman rank correlation analysis to obtain correlation coefficients ρ(VSD,LAIS) and significance values.

## 3. Results

**Figure 1:**
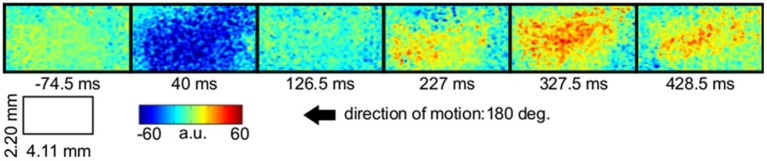
**Local active information storage (LAIS) allows to trace neural information processing in space and time**. Spatio-temporal structure of LAIS in cat area 18—seven frames from the spatio-temporal LAIS data, taken at the times indicated below each frame. Stimulation onset was at time 0. Baseline activity (−74.5 ms) is around zero and mostly uniform. At 40 ms after stimulus onset, LAIS is negative in a region that correlates to the region that later exhibits high LAIS. Around 227 ms increased LAIS sets in and lasts until the end of the data epoch, albeit with slow fluctuations (up to 1 s, see Figure 3). Also see the post-stimulus time-average in Figure 2.

**Figure 2:**
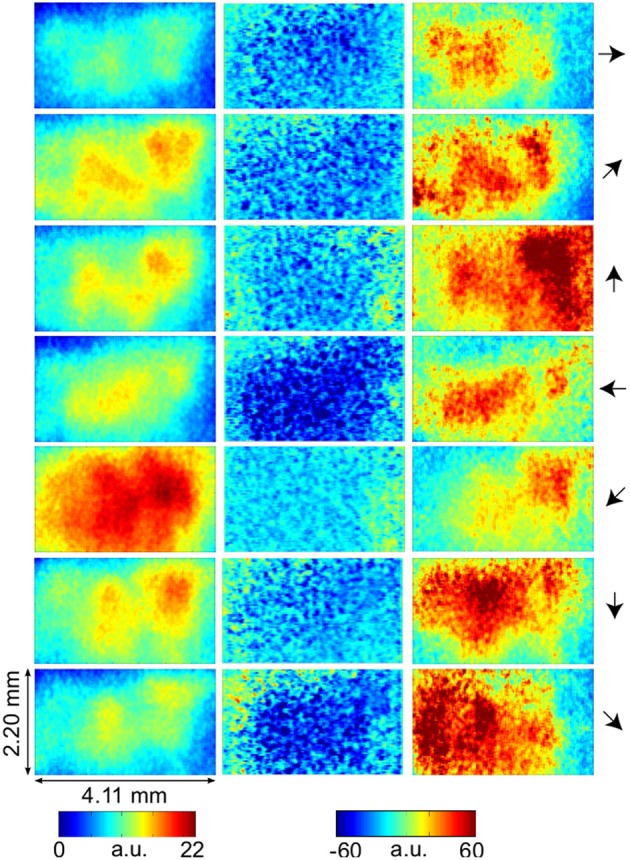
**VSD-activity and local active information storage (LAIS) maps**. VSD activity averaged over stimulation epochs and time after stimulus onset after the initial transient (0.2–1 s) (left column). LAIS map immediately after stimulus onset—negative values (blue) indicate surprise of the system (middle column). Time-average LAIS maps from the stimulus period after the initial transient (0.2–1 s) (right column). Rows 1–7 present different stimulus motion directions: 0, 45, 90, 180, 225, 270, 315 (in degrees, indicated by arrows on the right, arrow colors match time-trace colors in Figure 3). 67 × 137 data pixel per image, pixel dimension 30 × 32 μm^2^. Left–right image direction is anterior–posterior direction.

**Figure 3:**
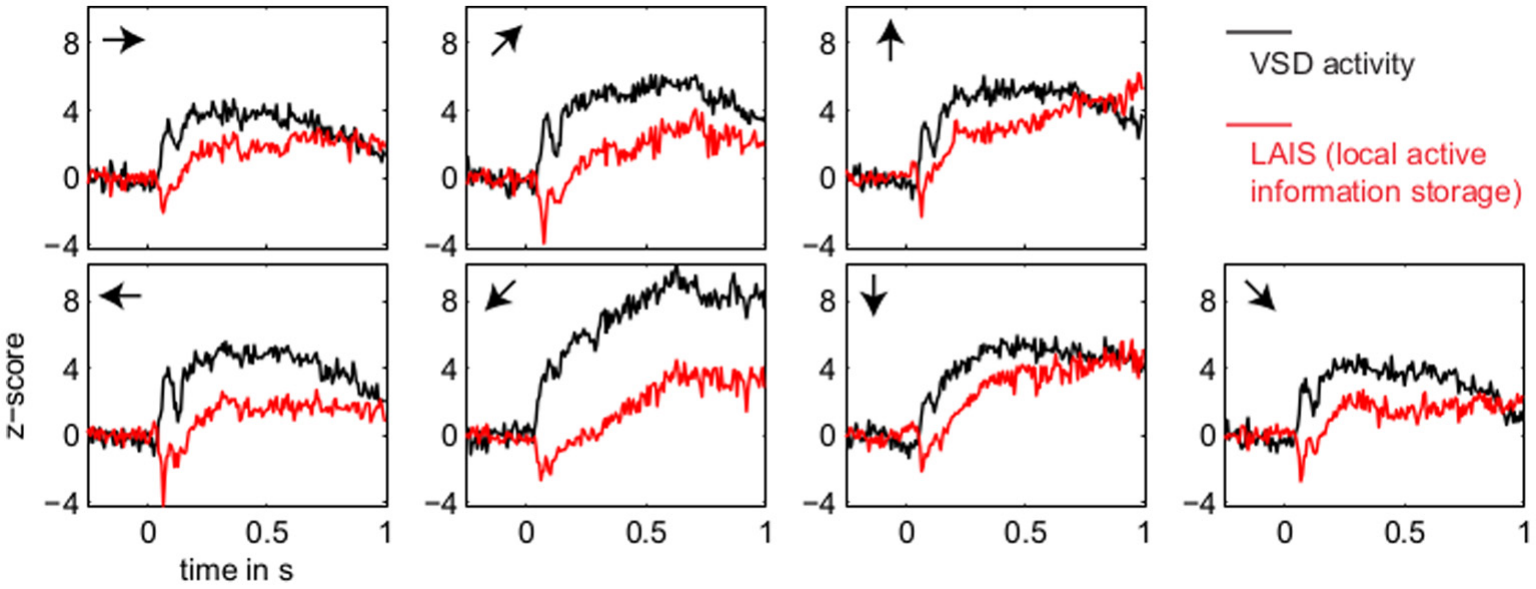
**Temporal evolution of VSD activity and local active information storage**. Spatial averages over the 67 × 137 data pixels for VSD activity (black traces), and the LAIS (red traces) versus time. Motion directions are indicated by arrows for each panel. Note that LAIS for the vertical, the right, and the downward-right motion directions continues to rise toward the end of the stimulus interval, despite declining activity levels. Also note that the unexpected onset response at approximately 40 ms leads to negative active information storage. For an explanation see the Materials and Methods section.

LAIS values exhibited a clear spatial and temporal pattern. The temporal pattern exhibited higher LAIS values during stimulation with a moving random dot pattern than under baseline stimulation with an isoluminant gray screen, with effects being largest in spatially clearly segregated regions (Figures 1–3). The spatial pattern of LAIS under stimulation was dependent on the motion direction of the drifting random dots in the stimulus (Figure 2).

In contrast to this spatially highly selective elevation of LAIS values under stimulation, there was a sharp drop in LAIS values at approximately 40 ms after stimulus onset, with negative LAIS values measured at many pixels (Figure 1, 40 ms window; Figure 2, middle column; Figure 3, lower row). This indicates that the baseline activity was misinformative about the following stimulus related activity (since an observer would expect the baseline activity to continue). This transient, stimulus induced drop in LAIS was more evenly distributed throughout the imaging window than the elevated LAIS in the later stimulus period post 200 ms (Figure 2, middle column). The transient drop in LAIS had a recovery time of approximately 34 ms, also giving an estimate of the dominant intrinsic storage duration of the neural processes.

In all conditions we observed a positive, but weak correlation between the local VSD activity values and LAIS values over time and space (Table 1). Looking at individual time intervals, we found stronger, and negative, correlation coefficients both, for the baseline interval (−1 to 0 s), and for the initial interval after the onset of the moving dot stimulus (0.04–0.14 s). In contrast, we observed a strong positive correlation at the late stimulus interval (0.2–1 s). This means that the increased dynamic range observed in the VSD signals during stimulation with the moving stimuli led to an increased amount of predictable information, rather than to a decrease. This correlation also means that storage was generally higher in neurons that were preferentially activated by the respective moving stimulus (also compare left and right columns in Figure 2 for each motion direction).

**Table 1:** **Correlation of LAIS and local VSD activity**.

**Motion direction**	**Correlation coefficient**			
	**Full epoch**	**−1 to 0 s**	**0.04–0.14 s**	**0.2–1 s**
0°	0.05^*^	−0.33^*^	−0.09^*^	0.45^*^
4 °	0.09^*^	−0.50^*^	−0.20^*^	0.65^*^
90°	0.12^*^	−0.30^*^	−0.13^*^	0.48^*^
180°	0.07^*^	−0.27^*^	−0.22^*^	0.44^*^
225°	0.07^*^	−0.58^*^	−0.22^*^	0.71^*^
270°	0.17^*^	−0.39^*^	−0.33^*^	0.68^*^
315°	0.03^*^	−0.37^*^	−0.17^*^	0.40^*^

*Correlation coefficients are Spearman rank correlations*.

* *p < 0.05/7*.

## 4. Discussion

Our results demonstrate increased local active information storage in the primary visual cortex of the cat under sustained stimulation, compared to baseline. The spatial pattern of the LAIS increase was clustered spatially and stimulus-specific (Figure 2). The temporal pattern of LAIS consisted of a first sharp drop in LAIS from 0.04 to 0.14 s after onset of the moving stimulus and a sustained rise in LAIS up to the end of the stimulation epoch (Figure 3). The sharp drop at stimulus onset for many pixels is important because it indicates the past activity of the pixels was surprising or misinformative about the next outcomes near that onset. This has the potential to be used in detecting changes of processing regimes directly from neural activity.

The subsequent sustained rise in LAIS is particularly notable because of the *random spatial* structure of each stimulus on a local scale; this random spatial structure translates into a random temporal stimulation sequence in the receptive field of each neuron because of the stimulus motion. The increased LAIS despite random stimulation of the neurons suggests that our observation is not due to input-driven storage, i.e., memory or storage contained already in the spatio-temporal stimulus features that drive the observed LAIS [as discussed in section 2.2.3 and by Obst et al. (2013)]. Nevertheless, as revealed by correlation analysis, storage was highest in regions preferentially activated by the stimulus, suggesting a representational nature of LAIS in these data with respect to the motion features of the stimulus. In sum, the changes of LAIS with stimulation onset, stimulation duration, and stimulus type clearly demonstrate that LAIS reflects neural processing, rather than mere physiological or instrumentation-dependent noise regularities. This leads us to believe that LAIS is a promising tool for the analysis of neural data in general, and of VSD data in particular.

### 4.1. Local active information storage and neural activity levels

Any increase in LAIS may in principle arise from two sources: first, a richer dynamics with a larger amplitude range—increasing overall information content, while maintaining the predictability of the time series (e.g., quantified as the inverse of the signal prediction error, or the entropy-normalized LAIS), may increase LAIS. Alternatively, increased LAIS may be based on increased predictability under essentially unchanged dynamics. The significant positive correlation between LAIS and VSD activity after stimulus onset suggests that a richer, but still predictable, dynamics of VSD activity is at the core of the stimulus-dependent effects observed here. As a caveat we have to note that the use of a kernel estimator for LAIS measurement, coupled with pooling of observations over the whole ensemble of pixels and time points may also have introduced a slight bias in favor of a positive correlation between high VSD activity and LAIS, as it allows storage to be more easily measured in pixels with larger amplitude here. The negative correlation observed in the baseline interval, however, demonstrates that this bias is not a dominant effect in our data. This is because a dominant effect of the kernel-based bias would also assign higher storage values to high amplitude data in the baseline interval, and thereby result in a positive correlation in the baseline. This was not the case. The relatively low correlation coefficients across the complete time-interval, which are between 0.02 and 0.13, further suggest that LAIS increases due not follow higher VSD signals tightly. Therefore, LAIS extracts additional useful information about neural processing. This point is further supported by the stimulus-dependent changes that seem more pronounced in LAIS maps than in the VSD activity maps (compare left and right columns in Figure 2).

For future studies the amplitude-bias problem introduced by the fixed-width kernel estimator should easily be overcome using a Kraskov-type variable width kernel estimator—see the original work of Kraskov et al. (2004), and Lindner et al. (2011); Vicente et al. (2011); Wibral et al. (2011, 2013); Lizier (2012) for implementation details of Kraskov-type estimators. Another possibility would be to condition the analysis on the activity level, as for example done for the transfer entropy measure by Stetter et al. (2012).

### 4.2. Timescales of LAIS

The recovery time of the stimulus-induced, transient drop in LAIS was 34 ms. A drop of this kind means that the activity before the drop (baseline activity) was not useful to predict the activity during the drop (the onset response). This is expected as the stimulus is presented in an unpredictable way to the neural system. However, the recovery time of this drop of approximately 34 ms yields an insight into the intrinsic storage time scales of the neural processes. We note that the observed time-scale corresponds to the high beta frequency band around 29 Hz (1/34 ms). In how far this is an incidental finding or bears significance must be clarified in future studies.

### 4.3. On the interpretation of local active information storage measures in neuroscience

When working with measures from information theory, it is important to keep in mind that the basic definition of information as given by Shannon revolves around the probabilities of events and the possibility to encode something using these events. To separate Shannon information content from information about something (new) in a more colloquial sense, one often also speaks about *potential* or *syntactic* information, when referring to Shannon information content, of *semantic* information when referring to human interpretable information, and last of pragmatic information for our everyday notion of information as in “news” [for details see for example the treatment of this topic by Deacon (2010)]. In the same way, LAIS does not directly describe information that the neural system stores about things in the outside world—rather, it quantifies how much of the future (Shannon) information in the activity can be predicted from its past.

In fact, information in the neural system *about* something in the outside world would have to be quantified by some kind of mutual information between aspects of the outside world and neural activity, while information in the classic sense of semantic information represented symbolically (e.g., in books, and other media) would be even more complicated: theoretically it should be quantified as a mutual information between the medium containing the symbols and activity in the neural system, while additionally satisfying the constraint that this mutual information should vanish when conditioning on the states of the world variables represented by the symbols.

While this lack of a more semantic interpretation of LAIS may seem disappointing at first, the quantification of the predictable amount of information makes this measure highly useful in understanding information processing at a more abstract level. This is important wherever we have not yet gained insights into what (if anything) may be explicitly represented by a neural system. Moreover, the focus on predictability provides a non-trivial link between LAIS and current theories of brain function as pointed out below. Nevertheless, a use of the concept in neuroscience may have to take the properties of the receiving neuron or brain area into account to consider how much of the mathematical storage in a signal is accessible to neural information processing. To address this concern, we used a pooling over all available data in space and time here as it seems to represent a way by which a receiving brain area could construct its (implicit) guesses of the underlying probability densities. However, also other strategies are possible and need to be explored in the future. As one example for another strategy of probability-density estimation, we have investigated a construction of probability densities via pooling over all data pixels but separately for each point in time. This approach avoids any potential issues with non-stationarities, but obscures the view of the “typical transitions” in the system over time to a point that no interpretable results were obtained (data not shown).

### 4.4. Local active information storage and predictive coding theories

Information storage in neural activity means that information from the past of a neural process will predict some non-zero fraction of information in the future of this process. It is via this predictability improvement that information storage is also tightly connected with predictive coding, an important family of theories of cortical function. Predictive coding theories propose that a neural system is constantly generating predictions about the incoming sensory input (Rao and Ballard, 1999; Knill and Pouget, 2004; Friston, 2005; Bastos et al., 2012) to adapt internal behavior and processing accordingly. These predictions of incoming information must be implemented in neural activity, and they typically need to be maintained for a certain duration—as it will typically be unknown to the system when the predictive information will be needed. Hence, the neural activity subserving prediction must itself have a predictable character, i.e., non-zero information storage *in activity*. Analysis of active information storage may thereby enable us to test central assumptions of predictive coding theories rather directly. This is important because tests of predictive coding theories so far mostly relied on the predictions being explicitly known and then violated—a condition not given for most brain areas beyond early sensory cortices, and for most situations beyond simple experimental designs. Here, the quantification of the predictability of brain signals themselves via LAIS may open a second approach to testing these important theories. To this end we may scan brain signals for negative LAIS, as negative LAIS values indicate the past states of the neural signals in question were not informative about the future, i.e., negative LAIS signals a breakdown of predictions. In our example dataset this was brought about by the sudden, unexpected onset of the stimulus. However, the same analyses may be applied in situations that are not a under external control—for example to analyze internally driven changes in information processing regimes.

In relation to predictive coding theories it is also encouraging that the predictive information was found on timescales related to the beta band. This is because this frequency band has been implied in the intra-cortical transfer of predictions (Bastos et al., 2012).

### 4.5. Sub-sampling and coarse graining, and non-locality of PDF estimation

When interpreting LAIS values it should be kept in mind that in neural recordings we typically do not observe the system fully or at the relevant scales—in contrast to artificial systems, such as cellular automata and robots, where the full system is accessible. More precisely, in neural data one of two types of sub-sampling is typically present—either coarse graining with local averaging of activity indices (as in VSD) or sub-sampling proper, where neural activity is recorded faithfully (e.g., via intracellular recordings) but with incomplete coverage of the full system. This sub-sampling may have non-trivial effects on the probability distributions of neural events [see for example Priesemann et al. (2009, 2013)]. Hence, LAIS values obtained under sub-sampling should be interpreted as *relative* rather than absolute measures and should only be compared to other experiments, or experimental conditions, when obtained under identical sampling conditions.

In addition there is necessarily temporal subsampling in the form of finite data; we therefore note again the potential for bias in the actual MI values returned via the use of kernel estimation here, particularly for large embedding dimensions and small kernel widths. Alternatives to kernel estimator are known to be more effective in bias compensation [e.g., Kraskov-Grassberger-Stögbauer estimation (Kraskov et al., 2004)]; or use of use kernel estimation is solely motivated by practical computational reasons. Effects of temporal subsampling also mandates to focus on relative rather than absolute values within this experiment.

Even within the experiment though, the bias may not be evenly distributed amongst the local MI values, which tend to exhibit larger bias for low frequency events. With that said, our experiment did use a large amount of data (by pooling observations over pixels and time), which counteracts such concerns to a large degree, and many of the key results (e.g., Figure 3) involve averaging or correlating over many local values, which further ameliorates this. There are techniques suggested to alleviate bias in local or pointwise MI, e.g., Turney and Pantel (2010), and while none were applied here, we do not believe this alters the general conclusions of our experiment for the aforementioned reasons. As a particular example, the surprise caused by the onset of stimulus is still clearly visible as negative LAIS, despite any propensity for such low frequency events to have been biased strongly toward positive values.

### 4.6. On the locality of information values

As a concluding remark, we would like to point out again that various “levels of locality” have to be carefully chosen in the analysis of neural data. One important level is the spatial extent (ensemble of agents) and the time span over which data are pooled to obtain the PDF. However, even pooling over a large spatial extent, i.e., many agents and a long time span, may still allow to interpret the information value of the data agent-by-agent and time step-by-time step, if agents *i* are *identical* and samples at subsequent time points *t* come from a *stationary* random process [see the book of Lizier (2013) for several examples]. This is because one may pool data to estimate a PDF as long as these data can be considered “replications,” i.e., as coming from the same random variable. Pooling data under these conditions will obviously not bias the PDF estimate away from the ground truth for any agent or time step. Irrespective of how many data points are pooled this way, it is then still possible to interpret each data point (*x*_*i,t*_, **x**^*k*−^_*i,t* − 1_) individually in terms of its LAIS, *a*(*x*_*t*_, **x**_**t**_^*k*−^_*t* − 1_). This locality of information values is identical to the local interpretation of the (Shannon) information terms *h*(*x*_*i*_) = −log(*p*(*x*_*i*_)) that together, as a weighted average over all possible outcomes *x*_*i*_, yield the (Shannon) entropy *H*(*X*) = ∑_*i*_ *p*(*x*_*i*_)*h*(*x*_*i*_) of a random variable *X*. As explained for example by MacKay (2003, chapter4), each and every outcome *x*_*i*_ of a random variable *X* has its own meaningful Shannon information value *h*(*x*_*i*_), that may be very different from that of another outcome *x*_*j*_, although repeated draws from this random variable can be considered stationary. It is this sense of “local” that gives *local* active information storage its name. In contrast, how locally in space and time we obtain the PDF is more important for the precision of the LAIS estimates.

In the analysis of LAIS from neural data three issues will necessarily blur locality, and impair the precision of the LAIS estimate to some extent:

1. If a pool of identical agents *i*, all running identical stationary random processes X_*i*_, is available, the only blurring of locality arises due to the intrinsic temporal extent of the state variables. However, the while the stored information may be encoded in a temporally non-local state **x**_**t**_^*k*−^_*t* − 1_, this information is used to predict the next value of the process *x*_*t*_ at a *single* point in time.
2. If agents are non-identical, but their data are pooled nonetheless, then the overall empirical PDF obtained across these agents is no longer fully representative of each single agent and the local information storage values per agent are biased due to the use of this non-optimal PDF. This effect may be present to some extent in our analysis, as we cannot guarantee that all parts of area 18 behave strictly identical.
3. If the random process in question is not stationary, then a PDF obtained via pooling samples across time is also not representative of what happens at single points in time, and again a bias in the LAIS values for each agent and time step arises. This bias is potentially more severe. Nevertheless, we pooled data across all available time samples here, as this seems to be closer to the strategy available to a neuron in a downstream brain area (also see section 2.2.5), when trying to estimate, or adapt to, its input distribution. This is because a neuron may more easily estimate approximate PDFs of its inputs across time than across all possible neurons in an upstream brain area, to most of which it simply doesn't interface.

## 5. Conclusion

Distributed information processing in neural systems can be decomposed into component processes of information transfer, storage and modification. Information storage can be quantified locally in space and time using an information theoretic measure termed local active information storage (LAIS). Here we present for the first time the application of this measure to neural data. We show that storage reflects neural properties such as stimulus preferences and surprise, and reflects the abstract concept of an ongoing stimulus despite the locally random nature of this stimulus. We suggest that LAIS will be a useful quantity to test theories of cortical function, such as predictive coding.

### Conflict of interest statement

The authors declare that the research was conducted in the absence of any commercial or financial relationships that could be construed as a potential conflict of interest.
